# Changes in mitochondrial stability during the progression of the Barrett’s esophagus disease sequence

**DOI:** 10.1186/s12885-016-2544-2

**Published:** 2016-07-19

**Authors:** N. J. O’Farrell, R. Feighery, S. L. Picardo, N. Lynam-Lennon, M. Biniecka, S. A. McGarrigle, J. J. Phelan, F. MacCarthy, D. O’Toole, E. J. Fox, N. Ravi, J. V. Reynolds, J. O’Sullivan

**Affiliations:** Trinity Translational Medicine Institute, Department of Surgery, Trinity College Dublin, St. James’s Hospital, Dublin 8, Ireland; Education and Research Centre, St. Vincent’s University Hospital, Elm Park, Dublin 4, Ireland; Trinity Translational Medicine Institute, Department of Clinical Medicine, Trinity College Dublin, St. James’s Hospital, Dublin 8, Ireland; Department of Pathology, University of Washington, Seattle, WA 98195 USA

**Keywords:** Barrett’s esophagus, Mitochondrial instability, Oxidative stress

## Abstract

**Background:**

Barrett’s esophagus follows the classic step-wise progression of metaplasia-dysplasia-adenocarcinoma. While Barrett’s esophagus is a leading known risk factor for esophageal adenocarcinoma, the pathogenesis of this disease sequence is poorly understood. Mitochondria are highly susceptible to mutations due to high levels of reactive oxygen species (ROS) coupled with low levels of DNA repair. The timing and levels of mitochondria instability and dysfunction across the Barrett’s disease progression is under studied.

**Methods:**

Using an in-vitro model representing the Barrett’s esophagus disease sequence of normal squamous epithelium (HET1A), metaplasia (QH), dysplasia (Go), and esophageal adenocarcinoma (OE33), random mitochondrial mutations, deletions and surrogate markers of mitochondrial function were assessed. In-vivo and ex-vivo tissues were also assessed for instability profiles.

**Results:**

Barrett’s metaplastic cells demonstrated increased levels of ROS (*p* < 0.005) and increased levels of random mitochondrial mutations (*p* < 0.05) compared with all other stages of the Barrett’s disease sequence in-vitro. Using patient in-vivo samples, Barrett’s metaplasia tissue demonstrated significantly increased levels of random mitochondrial deletions (*p* = 0.043) compared with esophageal adenocarcinoma tissue, along with increased expression of cytoglobin (CYGB) (*p* < 0.05), a gene linked to oxidative stress, compared with all other points across the disease sequence. Using ex-vivo Barrett’s metaplastic and matched normal patient tissue explants, higher levels of cytochrome c (*p* = 0.003), SMAC/Diablo (*p* = 0.008) and four inflammatory cytokines (all *p* values <0.05) were secreted from Barrett’s metaplastic tissue compared with matched normal squamous epithelium.

**Conclusions:**

We have demonstrated that increased mitochondrial instability and markers of cellular and mitochondrial stress are early events in the Barrett’s disease sequence.

## Background

Esophageal cancer is one of the most rapidly increasing malignancies in the Western world [1]. Overall 5-year survival rates are low at approximately 14 % [2]. The last several decades have seen a change in the histological trend of this malignancy with adenocarcinoma now representing the leading sub-type in the West [3]. Barrett’s esophagus is a pathologic precursor of esophageal adenocarcinoma. Following the classic metaplasia-dysplasia-adenocarcinoma sequence, it is speculated cancer development does not occur directly from non-dysplastic disease [4]. While Barrett’s esophagus follows this natural stepwise progression, the exact cellular instability mechanisms triggering cancer conversion are not fully elucidated.

The rate of mutagenesis in mitochondrial DNA is approximately 10-times higher than mutation rates in nuclear DNA. This is due to a combination of factors such as less proficient DNA repair mechanisms and high levels of exposure to ROS generated by the mitochondria themselves [5, 6]. The Warburg effect describes a phenomenon of altered energy metabolism in cancer cells, with increased lactate production despite sufficient oxygen to power oxidative phosphorylation [7]. This state of aerobic glycolysis does not arise in normal functioning cells and it is speculated that cancer cells divert from normal energy pathways as a protective mechanism to promote cell survival [8, 9]. These factors of increased susceptibility to DNA injury, along with evidence of altered energy metabolism in the cancer setting, have highlighted the potential role of the mitochondria in human cancer development [10–12].

To date, research has primarily focussed on clonal mitochondrial mutations in relation to esophageal cancer [13]. Little work has explored the role of random mitochondrial point mutations as a trigger for Barrett’s cancer development. With each cell containing hundreds of mitochondria and correspondingly thousands of strands of the mitochondrial genome, it is possible for wild type and mutated mitochondrial DNA to co-exist [12]. It is hypothesized that during tumor development cancer cells exhibit a mutator phenotype, with higher frequencies of random mutations, which are not supported by the normal rates of mutagenesis seen in non-cancerous cells [14]. The mutator phenotype hypothesis proposes that cancer cells must incur increased rates of mutagenesis during disease progression [14, 15], and as such, this theory suggests that benign tumors with low levels of random mutations will not progress to malignancy. Lee et al. [16] have demonstrated significantly increased random mitochondrial mutations in Barrett’s specialized intestinal metaplasia (SIM) compared with adjacent normal tissue. It was hypothesized that instability within the mitochondria play a crucial role in Barrett’s cancer development, however, profiling these changes along the Barrett’s disease sequence remain largely unexplored.

With respect to Barrett’s esophagus, with less than 0.12 % of cases per year progressing to esophageal adenocarcinoma [17], it is prudent that we understand the early instability mechanisms involved in the cancer switch. The purpose of this study was to identify changes in mitochondrial mutation rates and function along the Barrett’s disease sequence using in-vitro, in-vivo and ex-vivo models.

## Methods

### In-vitro cell line sequence

Four esophageal cell lines were used; HET1A, QH, Go and OE33 cells representing the normal squamous epithelium-SIM-high grade dysplasia (HGD)-esophageal adenocarcinoma (EAC) sequence, respectively. HET1A cells were obtained from American Type Culture Collection (ATCC) (LGC Standards, Middlesex, UK), and maintained in antibiotic-free bronchial epithelial cell basal media (BEBM) enhanced with hormonal cocktail BEGM® SingleQuots®. QH and Go cells were obtained from ATCC and cultured in BEBM enhanced with BEGM® SingleQuots® and supplemented with 10 % foetal calf serum (FCS) (Lonza, MD, USA) and 1 % penicillin/streptomycin (Lonza). OE33 esophageal adenocarcinoma cells were sourced from the European Collection of Cell Cultures (Sailsbury, UK) and maintained in Roswell Park Memorial Institute (RPMI) 1640 medium (Lonza) supplemented with 10 % FCS and 1 % penicillin/streptomycin. All cell lines were maintained at 37 °C.

### Mitochondrial random mutation capture (RMC) assay in cell lines and patient tissue

All research was carried out in accordance with the Declaration of Helsinki, with all patients providing informed written consent, and approval for this study was granted by the St James’s Hospital and Adelaide, Meath and National Children’s Hospital Institutional Review Board. In vivo patient samples were snap frozen in liquid nitrogen and stored at −80 °C for RMC assay experiments. Patients with histologically confirmed SIM and Barrett’s-associated EAC were recruited while in attendance of Barrett’s esophagus surveillance endoscopy or esophagectomy at the National Esophageal and Gastric Centre at St. James’s Hospital, Dublin. In-vitro, HET1A, QH, Go and OE33 cells, of similar cell passage number were grown to 70–80 % confluence in 25 cm^2^ flasks to isolate DNA for the RMC assay. In order to examine mitochondrial instability, random mitochondrial point mutations and deletions were quantified using detailed methods previously published by our group [18]. This RMC assay allows the quantification of point mutations and deletions in the mitochondrial genome at single molecule resolution. All tissues were analyzed in a blinded fashion. All PCR products were sequenced by the High-Throughput Sequencing Facility at the University of Washington.

### Evaluation of mitochondrial function using mitochondrial assays for reactive oxygen species (ROS)

To further examine mitochondrial biology, ROS levels were examined in vitro. Cells were seeded in 96 well plates at density 2500 to 8000 cells/well, depending on the cell line. Seeding at different concentrations was necessary to compensate for different growth rates between different cell lines, in order to ensure the same degree of confluence at the initiation of functional experiments. Following 24 h, ROS levels were assessed. Cells were incubated with 5 μM 2, 7-dichlorofluorescein diacetate (Sigma-Aldrich) using our previously published protocols [18]. Following 40 min incubation, the ROS probe was removed, cells were analyzed using the Spectra Max Gemini System at excitation 485 nm and emission 538 nm. Mean fluorescence values for each cell line were obtained from at least three independent experiments. Crystal violet assays were performed at the same time to allow for normalization of ROS levels to cell number in each cell line. Following 24 h, the media was decanted. Cells were washed with PBS, fixed with 1 % gluteraldehyde (Sigma-Aldrich) for 20 min, 1 % gluteraldehyde was discarded and 0.1 % crystal violet solution was added for 30 min and removed by washing with water. Plates were blotted on tissue paper and allowed to air dry on the bench overnight. Once dry, the cells were resuspended in 1 % Triton X100 (Sigma-Aldrich) and incubated on a shaker for 15 min. The absorbance was read at 550 nm using a Perkin Elmer Wallac 1420 Victor2 plate reader.

### In-vivo Cytoglobin (CYGB), oxidative stress gene analysis

Cytoglobin (CYGB), a gene linked with oxidative stress [19], was assessed in samples from patients representing the Barrett’s disease sequence; normal squamous epithelium (*n* = 6), SIM (*n* = 30), low-grade dysplasia (LGD) (*n* = 6), HGD (*n* = 12) and EAC (*n* = 8). Expression of this gene was used as a proxy marker for oxidative or cellular stress, with increased ROS levels associated with an increase in CYGB gene expression, in order to scavenge excess ROS [20–22]. The purpose of this experiment was to allow us to perform an in-vivo cellular stress assessment to complement our in-vitro ROS analysis. All cases were prospectively recruited at our national referral centre. Following each biopsy for standard histological examination, a matched biopsy for RNA extraction was immediately taken directly adjacent. Matched biopsies were placed in RNAlater preservative solution (Invitrogen) and transferred to the laboratory. Normal control samples were taken from individuals attending for upper GI endoscopy with no evidence of gastro-esophageal reflux or other inflammatory aetiology. If the esophagus was macroscopically normal at endoscopy, biopsies were taken and stored as above. Only samples which demonstrated normal squamous mucosa were used in further analysis. Cancer samples were taken from individuals undergoing assessment for a new diagnosis of EAC arising in a setting of Barrett’s esophagus. All cancer cases were chemotherapy and radiotherapy naïve. All histological examination was carried out by GI pathologists. All samples were placed immediately in RNAlater at the time of endoscopy. Samples were transferred to a 4 °C fridge overnight. The following day all samples were transferred to a −20 °C freezer for storage pending the results of histological examination of matched tissues. Following histological examination of matched biopsies, samples were selected for analysis across the different histological groups.

Patient material was homogenized using a Tissue-Lyser for 5 mins at a frequency of 25 pulses per second and RNA was extracted using the Qiagen Rneasy Mini Kit (Qiagen). RNA quantity and quality was determined spectrophotometrically using a Nanodrop 1000 spectrophotometer (NanoDrop, Technologies, Wilmington, DE) and quality assessment of all samples was performed on the Agilent 2100 bioanalyzer platform (Agilent technologies, Santa Clara, CA), using the RNA Nano 6000 kit. High quality total RNA was reverse transcribed to cDNA using random hexamer oligodeoxyribonucleotides that prime mRNA for cDNA synthesis. Quantitative PCR was used to quantify cytoglobin mRNA expression (ABI Biosystems) in samples relative to the 18S ribosomal RNA endogenous control and analyzed using SDS 2.3 and SDS RQ Manager 1.2 relative quantification software. Analysis of gene expression data was performed using the 2^ΔΔCt^ relative quantification method using the change in the expression of a target gene relative to the expression of a reference sample in the study.

### Measurement of secreted mitochondrial proteins and inflammatory cytokines from ex-vivo Barrett’s and matched normal explant tissue

Barrett’s esophagus patients’ biopsies (*n* = 12), from areas of SIM and surrounding normal tissue, were obtained for fresh ex-vivo explant culture at 37 °C. Matched-normal tissue biopsies were taken ≥ 5 cm from the most proximal border of macroscopic Barrett’s. Biopsies were immediately placed on saline-soaked gauze and transported within 10 min to the laboratory for ex-vivo culture 24-well plates containing 1 mL of M199 media (Lonza) supplemented with 10 % FCS, 1 % penicillin/streptomycin and 1 μg/ml insulin. Tissues were cultured for 24 h, and conditioned media stored at −80 °C. Barrett’s tissue was characterized by examining for the expression of the columnar epithelium molecular markers cytokeratin 8 and villin (Metabion). Tissue viability following explant culture was confirmed using a lactate dehydrogenase (LDH) assay (Caymanchem, Michigan, USA). Secretions of surrogate mitochondrial proteins, cytochrome c and SMAC/Diablo were measured in explant conditioned media using commercially available enzyme-linked immunosorbent assay (ELISA) kits (R&D Systems) using protocols as per manufactures’ instructions. The levels of cytokines interleukin-8 (IL-8), interleukin-6 (IL-6), interleukin-1β (IL-1β) and tumour necrosis factor-α (TNF-α) were measured using a multiplex assay from Mesoscale Discovery® (Gaithersburg, MD, USA) using protocols as per manufactures’ instructions.

### Statistical analysis

Data were analyzed with SPSS (PASW [Predictive Analytics Software] version 18) (IBM, Armonk, New York, USA) and Graph Pad Prism (Graph Pad Prism, San Diego, CA) software. Differences between HET1A, QH, Go and OE33 cell lines were calculated using unpaired Student’s t-tests and Kruskal Wallis tests. In-vitro results were reported as mean and variation was expressed as standard deviation (SD). In-vivo and ex-vivo: differences between continuous variables, for matched patient groups were calculated using Wilcoxon signed-rank tests, while in unmatched patient groups the Mann–Whitney *U* test was used. Patient data were reported as mean and variation was expressed as standard error of the mean (SEM = SD divided by the square root of the sample size). Statistical significance was defined as *p* ≤ 0.05.

## Results

### Mitochondrial instability levels in the Barrett’s disease model

#### In-vitro cell line assessment

**Fig. 1:**
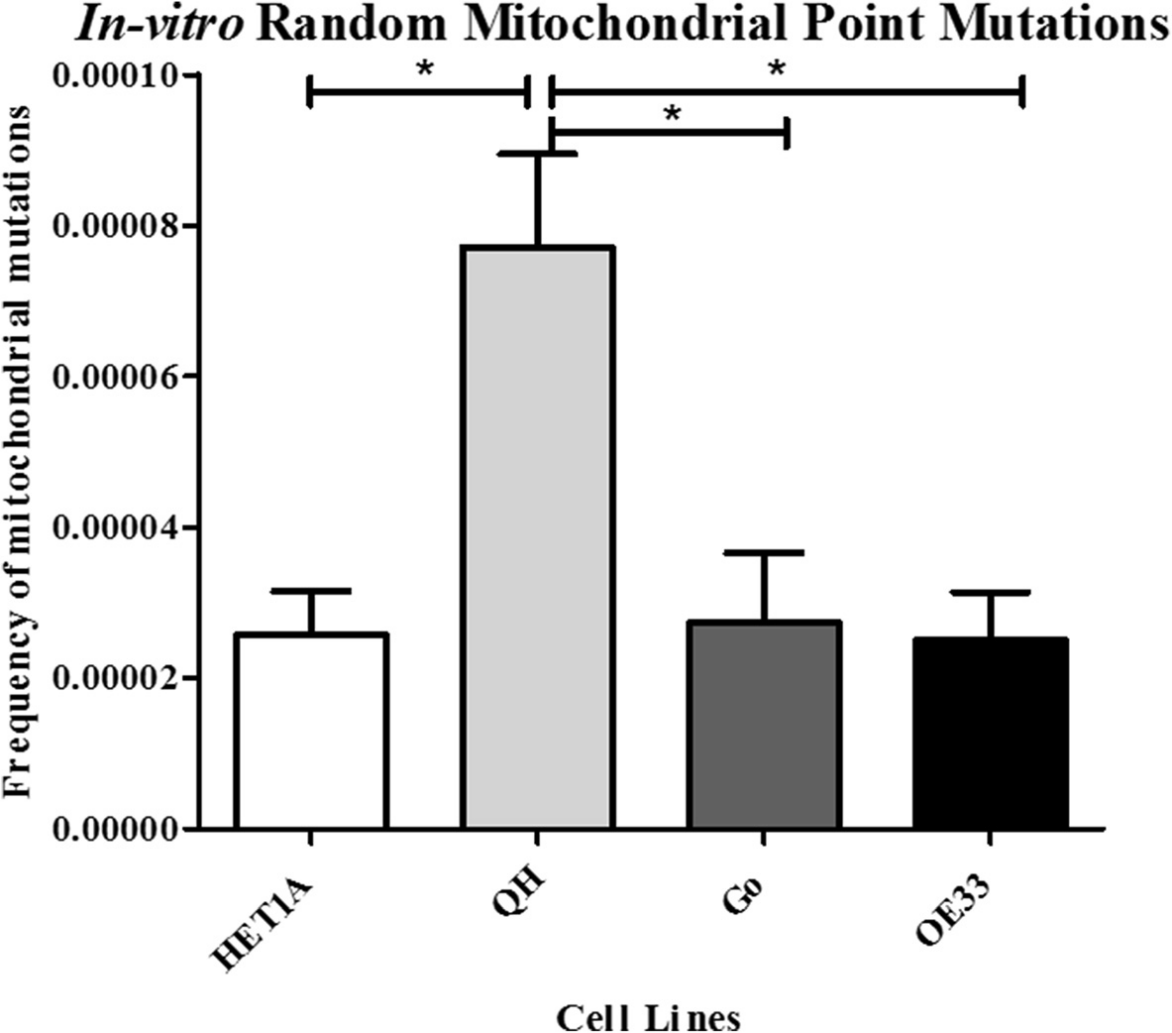
Random mitochondrial point mutations in-vitro. There was a significantly increased frequency of random mitochondrial DNA mutations in the QH cells (mean 7.710 × 10^−5^, SD 2.770 × 10^−5^) (*n* = 5) compared to HET1A (mean 2.560 × 10^−5^, SD 1.015 × 10^−5^) (*n* = 3), Go (mean 2.730 × 10^−5^, SD 2.440 × 10^−5^) (*n* = 5) and OE33 (mean 2.500 × 10^−5^, SD 1.430 × 10^−5^) (*n* = 5) cells. This demonstrated that random mutations were an early event in this in-vitro model of Barrett’s progression. *p ≤ 0.05

Significantly elevated random mitochondrial point mutations were an early event in the Barrett’s in-vitro model (Fig. 1). Levels of random mitochondrial point mutations were significantly increased in the QH, metaplastic cells, compared to the HET1A cells (*p* = 0.024), Go cells (*p* = 0.008) and OE33 cells (*p* = 0.006). No significant difference was demonstrated in the frequency of random mitochondrial mutations between the HET1A, Go and OE33 cell lines (all *p* values >0.05). Levels of mitochondrial deletions were not evident in-vitro.

#### In-vivo patient tissue assessment

**Fig. 2:**
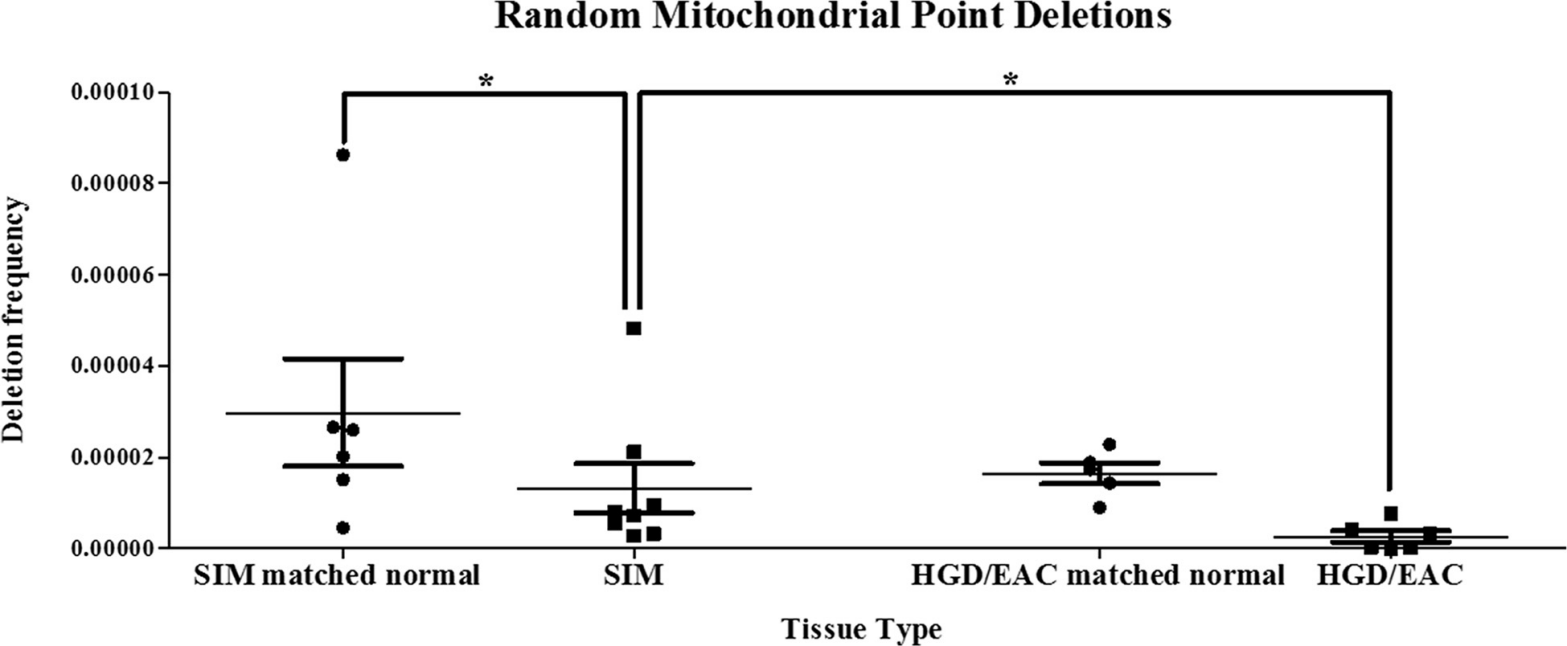
Random mitochondrial point deletions in-vivo. Wilcoxon matched-paired signed rank tests demonstrated a significantly increased level of deletions in the SIM matched normal tissue compared with SIM (*p* = 0.031) and a trend towards increased deletions in HGD/EAC-matched normal tissue compared with areas of HGD/EAC (*p* = 0.063). Mann Whitney-*U* test demonstrated significantly increased frequencies of deletions in SIM compared to HGD/EAC tissue (p=0.043). *p ≤ 0.05

While there were no differences in the frequency of random mutations between SIM (mean = 8.269 × 10^−5^, SEM = 2.223 × 10^−5^) and HGD/EAC tissue (mean = 7.422 × 10^−5^, SEM = 1.615 × 10^−5^) (*p* = 1.00) (data not shown), interestingly, random mitochondrial deletions were significantly increased in SIM tissue (mean = 1.322 x 10^-5^ SEM = 5.400 x 10^−6^) compared with HGD/EAC (mean = 2.63 x 10^−6^, SEM = 1.250 × 10^−6^) (*p* = 0.043) (Fig. 2). Random deletions were significantly increased in SIM matched-normal tissue (mean = 2.983 × 10^−5^, SEM = 1.178 × 10^−5^) compared with SIM biopsies (*p* = 0.031) (Fig. 2). While not significant, there was a trend towards increased mitochondrial deletions in matched-normal tissue (mean = 1.652 × 10^−5^, SEM = 2.331 × 10^−6^) compared with HGD/EAC cancerous tissue (*p* = 0.063).

**Fig. 3:**
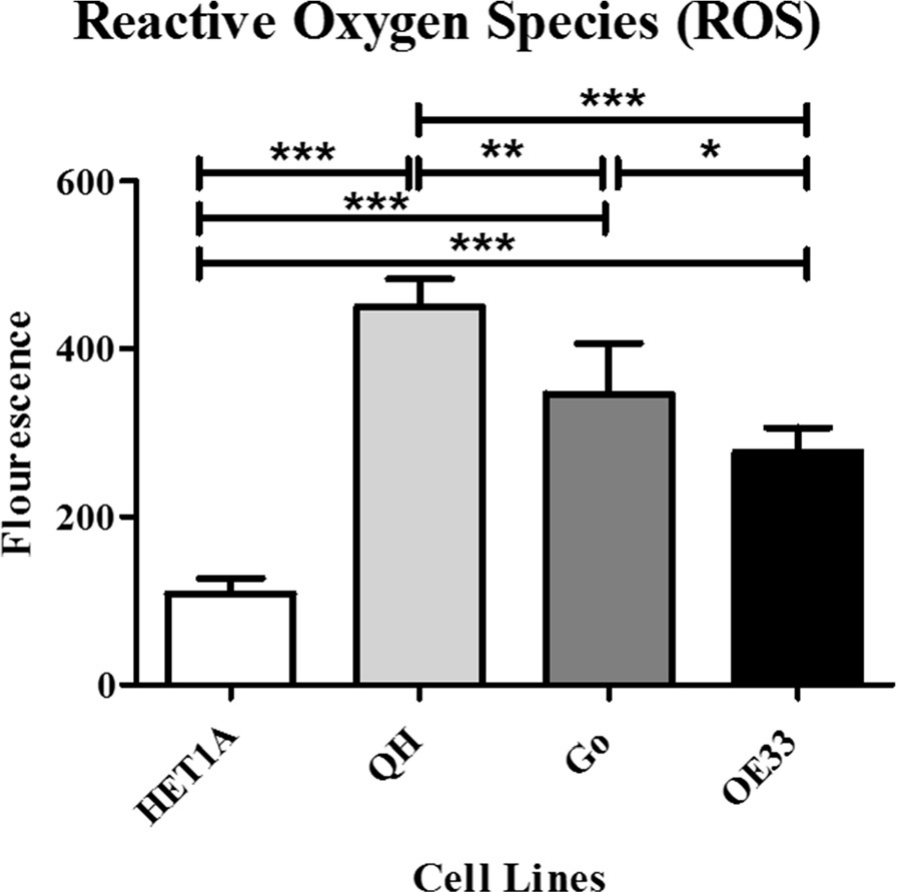
Mitochondrial function, ROS levels, across the Barrett’s disease sequence (*n* = 5). ROS was significantly lowest in the HET1A cells and highest in the QH cells. ROS levels were significantly increased in the QH cells compared with Go (*p* = 0.003) and OE33 (*p* < 0.0001) cell lines. ROS levels were 1.3 times higher in the Go cell line compared with the OE33s (*p* = 0.020). *p ≤ 0.05, **p ≤ 0.005, ***p ≤ 0.0005

### In-vitro Reactive Oxygen Species (ROS) assessment

There was a 4.2-fold increase in ROS in the QH cells (mean 449.77, SD 26.848) (*p* < 0.0001), a 3.2-fold increase in the Go cells (mean 346.4, SD 48.262) (*p* < 0.0001) and a 2.6-fold increase in the OE33 cells (mean 276.826, SD 23.188) (*p* < 0.0001), relative to the HET1As (mean 108.239, SD14.875). ROS levels were significantly higher in the QH cells compared with all other points in the Barrett’s cell line progression model (Fig. 3).

### Cytoglobin (CYGB) gene expression levels across the Barrett’s disease progression model

**Fig. 4:**
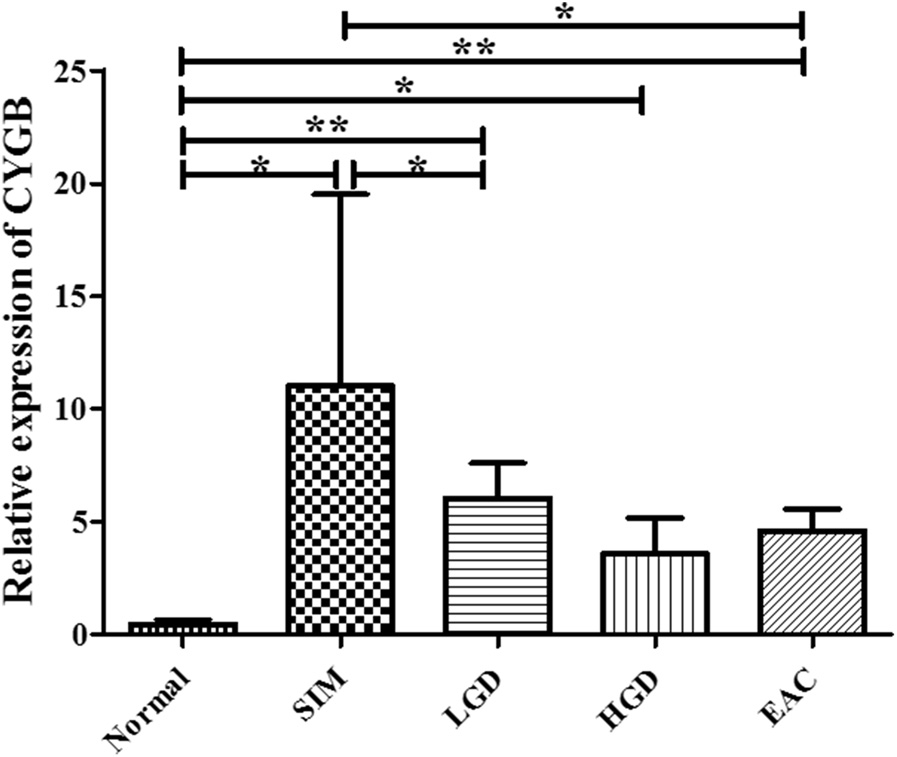
Expression of CYGB along the Barrett’s disease sequence. The expression of CYGB is demonstrated in normal (mean 0.425, standard error of mean [SEM] 0.231), SIM (mean 11.013, SEM 8.493), LGD (mean 6.03, SEM 1.555), HGD (mean 3.580, SEM 1.580) and EAC biopsies (mean 4.581, SEM 0.991). SIM over-expressed CYGB relative to LGD and EAC. There was a significant increase in CYGB in SIM, LGD, HGD and EAC samples when compared with normal squamous epithelium. *p < 0.05, **p < 0.005

There was a 25.9-fold increase in CYGB expression in SIM (mean 11.013, SEM 8.493) compared with normal biopsies (mean 0.425, SEM 0.231) (*p* = 0.013). Levels of CYGB was significantly increased in SIM cases compared to LGD (mean 6.03, SEM 1.555) (*p* = 0.010) and EAC (mean 4.581, SEM 0.991) (*p* = 0.022) (Fig. 4).

### Ex-vivo secretions of mitochondrial proteins and inflammatory cytokines from Barrett’s and matched normal explants

**Fig. 5:**
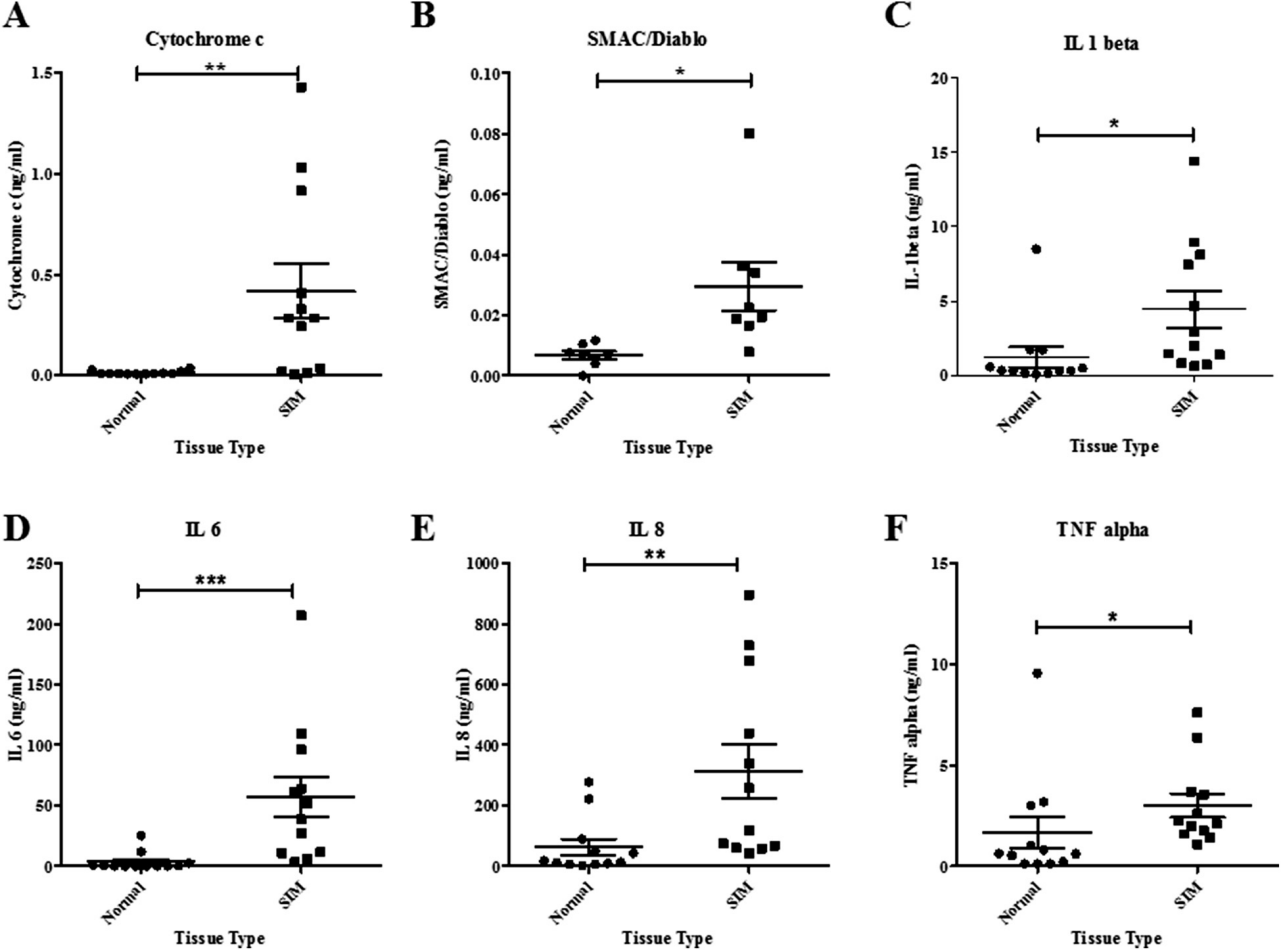
**a**-**f** Mitochondrial proteins and inflammatory cytokines levels in explant cultured media in SIM tissue and surrounding matched-normal tissue. Wilcoxon matched-pairs signed rank tests demonstrated significantly increased levels of **a** cytochrome c (n=12), **b** SMAC/Diablo (n=8), **c** IL-1beta (n=12), **d** IL-6 (n=12), **e** IL-8 (n=12) and **f** TNF-alpha (n=12) in SIM tissue compared to surrounding normal epithelium. *p ≤ 0.05, **p ≤ 0.005 and ***p ≤ 0.0005

Secreted cytochrome c and SMAC/Diablo were significantly higher from SIM tissue compared with matched normal tissue (*p* = 0.003, *p* = 0.008 respectively) (Fig. 5a, b). Inflammatory cytokines were also significantly increased in SIM tissue compared with matched normal tissue; IL-1β (*p* = 0.007), IL-6 (*p* = 0.0005), IL-8 (*p* = 0.002) and TNF-α (*p* = 0.034) (Fig. 5c-f).

## Discussion

The role of mitochondrial instability in the progression of Barrett’s esophagus is poorly understood. Metabolic imbalances, such as reduced response to apoptosis and increased glycolysis are all features of cancer cells, and are tightly regulated by the mitochondria [11, 23, 24]. Mutagenesis is a catalyst for cancer development, but to date, clonal gene mutations have been the main type of mitochondrial mutations analyzed with respect to esophageal carcinoma. The mitochondrial genome is more vulnerable to random mutations due to high ROS exposure and lower DNA repair mechanisms compared with nuclear DNA [5, 25]. Here we examined alterations in random mitochondrial point mutations/deletions and other markers of mitochondrial instability in the Barrett’s esophagus disease sequence using in-vitro, in-vivo and ex-vivo models.

Using an in-vitro cell line model, we demonstrated random mitochondrial mutations were significantly elevated in the metaplastic, QH, cells compared with all other points along the Barrett’s disease sequence, represented by the different cell lines. During tumor development, cancer cells are understood to exhibit a mutator phenotype with increased rates of mutagenesis during disease progression [14, 15, 26–28]. In this theory, benign tumors with low levels of random defects will not progress to malignancy, and the mutator frequency will influence risk. In other studies, increased random mitochondrial mutations have been reported in SIM compared with adjacent normal tissue [16]. In our study, using the RMC assay, mitochondrial deletions, a form of rearrangement of the mitochondrial genome and a recognized marker of mitochondrial instability [29], were significantly increased in SIM compared with HGD/EAC. An increased frequency of deletions in SIM compared with HGD/EAC is mirrored in colorectal polyp/cancer studies [30], both supporting the hypothesis that random mutations/deletions may become redundant as the disease progresses. It is recognized once malignant cells become established, selection processes ensue, with more aggressive mutations surviving and undergoing subsequent replication, with clonal mutations/deletions and not random ones overtaking the initial catalyst for cancer development at this time in the disease sequence [15, 31]. In this study, surrounding normal tissue demonstrated increased deletions compared with areas of SIM or HGD/EAC, suggesting mitochondrial instability is not just confined to the visible site of pathological tissue abnormality in the esophagus with Barrett’s disease, but exerts a field effect, which has been previously demonstrated in colorectal tumors [30].

The changes in the mitochondrial environment across the Barrett’s disease sequence were further measured through assessment of proxy markers of cellular stress in-vitro and in-vivo. We have shown in-vitro that levels of ROS in the QH, metaplasia cell line were significantly elevated compared to other points along the Barrett’s disease sequence. The esophagus is redox-sensitive [32], but the role of oxidative stress across the Barrett’s spectrum is largely unknown. Mitochondria are the main source of ROS production, with excess levels of ROS associated with oxidative damage [33–35]. The Warburg effect theorizes cancer cells reprogram energy metabolism, reducing oxidative phosphorylation and ROS production, potentially decreasing injury to mitochondrial DNA [36, 37]; perhaps this may explain the significant reductions in ROS in our in-vitro model between the QH metaplastic cells and the Go and OE33 cells. The role of ROS as a precursor for cancer progression has been studied in many cancers. In breast cancer, BRCA-1, a tumor suppressor gene, has been shown to play a role in protecting against ROS damage; BRCA-1 mutations have subsequently been implicated in loss of redox balance with increased ROS, and may potentially drive cancer development [38].

Studies have shown that the gene cytoglobin, CYGB, is associated with ROS levels and induced in response to oxidative stress where it can try to act to scavenge excess ROS [20–22]. We have shown that CYGB was over-expressed in SIM compared to levels detected in normal, LGD and EAC tissue, supporting the concept that Barrett’s metaplasia is an environment of oxidative stress, and the pre-neoplastic tissue maybe more susceptible to oxidative damage compared to neoplastic tissue similar to what has been documented in the prostate [39]. Other studies have shown that CYGB overexpression in-vitro can induce protection from chemically-induced oxidative stress but this is only seen at non-physiological concentrations of cytoglobin [19]. Loss of CYGB expression in the latter stages of the disease potentially may reflect the inability to regulate oxidative stress, and loss of protection once tumor growth is firmly established [19, 40].

As it is not possible to assess the active secretion of mitochondrial and inflammatory proteins in fixed tissue, using an ex-vivo explant model, we assessed the secretion of mitochondrial proteins in metaplastic tissues, as the greatest levels of instability and cellular stress were observed at this pathological stage, and compared it to matched normal mucosa. The explant model system is superior to monolayer cell cultures as it encompasses the tissue microenvironment [41]. Ex-vivo studies demonstrated a significant increase in cytochrome c and SMAC/Diablo, pro-apoptotic mitochondrial proteins in SIM tissue compared with matched normal tissue, patterns previously seen in esophageal cancer cell lines [42]. The mitochondria play a critical role in cell apoptosis. Cytochrome c and SMAC/Diablo are apoptotic proteins, released into the cytosol in order to activate a series of caspases downstream. These findings suggest an increase in mitochondrial biogenesis at the Barrett’s metaplastic stage. Mitochondria have an important role in pro-inflammatory signalling; similarly, pro-inflammatory mediators may also alter mitochondrial function. In parallel with increases in mitochondria protein secretion from metaplastic tissue, there were increases in inflammatory cytokines, IL-1β, IL-6, IL-8 and TNF-α. This complements previous observations from our group demonstrating associations between inflammation and mitochondrial instability in another inflammatory condition [43]. These data reinforce the finding that mitochondrial instability, oxidative stress and inflammatory changes are early events in the Barrett’s disease sequence. Strategies aimed at targeting these processes may represent preventive and therapeutic interventions.

## Conclusions

We have shown that mitochondrial instability, oxidative stress and increases in mitochondrial and inflammatory protein production are activated early in the Barrett’s disease progression sequence. Although unclear whether mitochondrial dysfunction is the cause or consequence of these events, this study shows that SIM occurs in an environment of increased oxidative stress and mitochondrial instability.

## Abbreviations

ATCC, American type culture collection; BEBM, bronchial epithelial cell basal media; CYGB, cytoglobin; EAC, esophageal adenocarcinoma; FCS, foetal calf serum; HGD, high grade dysplasia; IL, interleukin; LGD, low grade dysplasia; RMC, random mutation capture; ROS, reactive oxygen species; RPMI, Roswell Park Memorial Institute; SD, standard deviation; SEM, standard error of mean; SIM, specialized intestinal metaplasia; TNF-α, tumour necrosis factor-α
